# PubMed-supported clinical term weighting approach for improving inter-patient similarity measure in diagnosis prediction

**DOI:** 10.1186/s12911-015-0166-2

**Published:** 2015-06-02

**Authors:** Lawrence WC Chan, Ying Liu, Tao Chan, Helen KW Law, SC Cesar Wong, Andy PH Yeung, KF Lo, SW Yeung, KY Kwok, William YL Chan, Thomas YH Lau, Chi-Ren Shyu

**Affiliations:** 1 Department of Health Technology and Informatics, Hong Kong Polytechnic University, Hung Hom Kowloon, Hong Kong; 2 Institute of Mechanical and Manufacturing Engineering, School of Engineering, Cardiff University, Cardiff, CF24 3AA UK; 3 Department of Diagnostic Radiology, University of Hong Kong, Pokfulam, Hong Kong; 4 Informatics Institute and Department of Computer Science, University of Missouri, Columbia, MO USA

## Abstract

**Background:**

Similarity-based retrieval of Electronic Health Records (EHRs) from large clinical information systems provides physicians the evidence support in making diagnoses or referring examinations for the suspected cases. Clinical Terms in EHRs represent high-level conceptual information and the similarity measure established based on these terms reflects the chance of inter-patient disease co-occurrence. The assumption that clinical terms are equally relevant to a disease is unrealistic, reducing the prediction accuracy. Here we propose a term weighting approach supported by PubMed search engine to address this issue.

**Methods:**

We collected and studied 112 abdominal computed tomography imaging examination reports from four hospitals in Hong Kong. Clinical terms, which are the image findings related to hepatocellular carcinoma (HCC), were extracted from the reports. Through two systematic PubMed search methods, the generic and specific term weightings were established by estimating the conditional probabilities of clinical terms given HCC. Each report was characterized by an ontological feature vector and there were totally 6216 vector pairs. We optimized the modified direction cosine (mDC) with respect to a regularization constant embedded into the feature vector. Equal, generic and specific term weighting approaches were applied to measure the similarity of each pair and their performances for predicting inter-patient co-occurrence of HCC diagnoses were compared by using Receiver Operating Characteristics (ROC) analysis.

**Results:**

The Areas under the curves (AUROCs) of similarity scores based on equal, generic and specific term weighting approaches were 0.735, 0.728 and 0.743 respectively (p < 0.01). In comparison with equal term weighting, the performance was significantly improved by specific term weighting (p < 0.01) but not by generic term weighting. The clinical terms “Dysplastic nodule”, “nodule of liver” and “equal density (isodense) lesion” were found the top three image findings associated with HCC in PubMed.

**Conclusions:**

Our findings suggest that the optimized similarity measure with specific term weighting to EHRs can improve significantly the accuracy for predicting the inter-patient co-occurrence of diagnosis when compared with equal and generic term weighting approaches.

**Electronic supplementary material:**

The online version of this article (doi:10.1186/s12911-015-0166-2) contains supplementary material, which is available to authorized users.

## Background

The huge amount of clinical data managed by the electronic health record (EHR) system potentiate case-based decision support where the reference cases are retrieved based on their similarity with the current case of interest [1, 2]. To measure the inter-patient similarity consistently, the feature vector model has been established by transforming the clinical information of EHRs, including laboratory test findings, medical images and diagnostic reports, to vector elements systematically [3–6].

The transformation of textual information, such as image findings, to feature vector requires the support of a medical ontology [5, 6]. Systematized Nomenclature of Medicine (SNOMED) Clinical Terms (CT) is a collection of clinical terms that are organized as concepts and linked in a hierarchy with “is-a” or inverse “is-a” relationships [7–10]. Concepts at a particular level of the hierarchical structure are selected as the feature concepts. The edge count along the path connecting a term in EHR and a feature concept in the “is-a” hierarchy represents their semantic distance [3–5, 11, 12]. The ontological feature vector contains numerical elements, each of which is inferred by integrating the semantic distances from all the EHR terms to a feature concept. It has been proved that the ontological vector model significantly outperforms the simple string matching in predicting inter-patient co-occurrence of subclinical disorder [12].

Euclidean distance and direction cosine are two commonly used similarity measures but preserve different properties. Direction cosine measures the similarity according to the angle between two feature vectors only but Euclidean distance considers the magnitudes of two vectors in addition to the angle. With such property, Euclidean distance is more sensitive to the absolute difference between two EHRs than direction cosine. For high dimensional vector model, they achieved similar accuracy in nearest neighbour queries. However, the direction cosine is more computationally efficient than Euclidean distance because the ontological vectors usually have a large number of zero elements in the information retrieval applications, expediting the computation of direction cosine. Identifying similar examination reports for diagnosis prediction requires exhaustive search in imaging examination database. As the database is assumed to host a huge number of eligible reports, the efficiency for computing the similarity score of an eligible report with the query report becomes very crucial.

The modified direction cosine (mDC) was developed by Chan et al. (2011) to preserve the advantageous properties of both Euclidean distance and direction cosine and extend the applications to low dimensional vector model [12]. In mDC, the feature vector is augmented by a regularization constant of unity to acquire the property of Euclidean distance and maintain the computational efficiency of direction cosine [12]. Numerical overflow that happens for direction cosine can be avoided because the length of the feature vector will never be close to zero due to the inclusion of regularization constant in mDC. However, it is still questionable if the performance of mDC can be optimized against different values of this regularization constant.

The feature concepts of the above-mentioned vector model were equally weighted. In fact, clinical terms are unequally associated to a particular disease. For example, hepatic necrosis and cirrhosis are common image findings in the computed tomographic scan of HCC patients. However, “hepatic necrosis” is more spatially associated with cell death phenomenon in the simultaneous growth of HCC than “cirrhosis” that reveals a fibrotic condition following cell death in HCC. Thus, term weighting, which has been well established in bioinformatics, should be applied to improve accuracy of semantic measure or remove unrelated terms [13, 14].

The disease of interest in this work is HCC, one of the ten most common cancers in the world [15]. Abdominal tomographic scan plays an important role in the diagnosis of HCC because the images can show patterns characterizing the pathophysiology of HCC [16]. Such patterns, after observed by radiologists, will be recorded as findings in the image examination report. In this work, two novel weighting approaches, namely generic term weighting and specific term weighting, are proposed to improve the performance of the ontological vector model in predicting inter-patient HCC co-occurrence. The performances of these two approaches were compared with reference to the baseline approach of equal term weighting, in which all feature concepts were equally weighted and the independent constant has already been optimized.

The generic and specific term weighting approaches were implemented based on the systematic search of PubMed, a huge database indexing biomedical journal articles. We assumed that a term is highly related to a particular disease if the chance for co-mentioning the term, the disease of interest and their synonyms in the abstracts of the articles is high. The highly weighted terms identified by this work can also be used to index the reports for reminding the clinicians of follow-up using other clinical tests.

## Methods

### Clinical data collection

Under the criterion that liver is the region of interest for HCC, 112 image reports of abdominal computed tomography examinations were collected retrospectively from the Radiology Departments of four local hospitals in Hong Kong. HCC or liver metastases were reported in 59 cases and no abnormality detected (NAD) in the other 53 reports. The age range of the patients was from 4 to 88 at the time of data retrieval. The patients were de-identified by using a randomly generated unique ID. The personal information, including name, identity card number, telephone number and address, were removed from the reports by third party clinical personnel before data were collected by the research team. Human Subject Ethics Approval has been obtained from the Hong Kong Polytechnic University (HSEARS20140710002).

### Image finding term extraction

The clinical terms of image findings related to HCC were identified and extracted manually from the reports by five practicing radiographers (Authors: APHY, KFL, SWY, KYK, WYLC). They learnt the structure, content and use of SNOMED CT from the Unified Medical Language System (UMLS). The extraction of clinical terms was supervised and validated by a radiologist (Author: TC) and two professorial staff with anatomy and radiography background (Authors: HKWL, TYHL). The definitions of clinical terms and their synonyms are standardized by SNOMED CT and unified to concepts by UMLS Terminology Services (license code: NLM-0315126310) where a unique concept ID is assigned to each concept. The concepts for all the extracted image finding terms were identified. For the equal and generic term weighting approaches, the identified concepts were mapped to the corresponding feature concepts according to SNOMED CT and the mapped feature concepts and their synonyms were used for weighting. For the specific term weighting approach, the extracted terms and their synonyms were used for weighting.

### Ontological vector model

The relationship between concepts is defined by the “is-a” hierarchical tree of SNOMED CT, which consists of levels of concepts. As the concepts of the extracted terms exist at different levels, the reports can be consistently compared if the extracted terms are projected to the concepts at a particular level, which are referred to feature concepts. The level-4 concepts were chosen as feature concepts in this work because level-4 provides an optimal classification granularity for accurate patient matching [12].

**Fig. 1:**
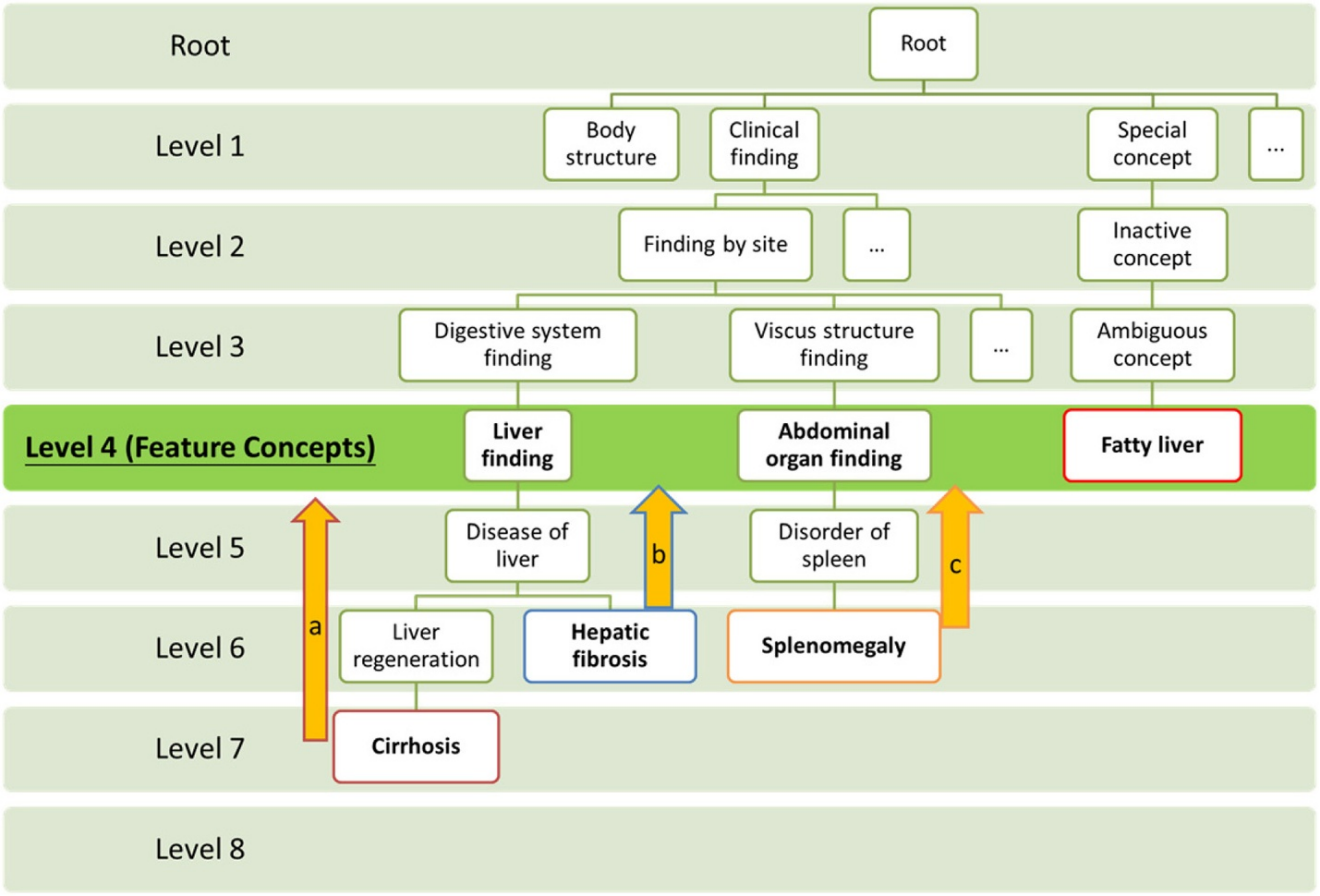
- Projection of image finding terms to feature concepts in SNOMED CT “is-a” hierarchy. Part of the “is-a” hierarchical relationships is illustrated with three examples demonstrating the rules to determine the semantic distances. Four image finding terms: “cirrhosis”, “hepatic fibrosis”, “splenomegaly” and “fatty liver” are considered. The level-4 concepts are regarded as feature concepts. In this case, feature concepts: “liver finding”, “abdominal organ finding” and “fatty liver” are involved. **a** The term “cirrhosis” at level-7 is the descendant of “liver finding”. Their semantic distance is 3 because there are three “is-a” links between them. **b** The semantic distance between “hepatic fibrosis” and “liver finding” is 2. **c** The term “splenomegaly” is not a descendant of “liver finding” but the descendant of “abdominal organ finding”. Thus, the semantic distance between “splenomegaly” and “liver finding” is infinity and that with “abdominal organ finding” is 2. Finally, the term “fatty liver” at level 4 is also a feature concept and the semantic distance is 0

Let *f*_*i*_, *m*, *d*_*j*_ and *n* be the *i*^th^ feature concept, the number of feature concepts, the *j*^th^ concept extracted from a report and the number of concepts extracted from the report respectively. The semantic distance between *f*_*i*_ and *d*_*j*_ is defined as *s*_*ij*_ ∈ [0,∞]. The value of *s*_*ij*_ is determined subject to three rules. (see Fig. 1)

1. If *d*_*j*_ is the descendant of *f*_*i*_, then *s*_*ij*_ is the number of “is-a” link from *d*_*j*_ to *f*_*i*_.
2. If *d*_*j*_ is not the descendant of *f*_*i*_, then *s*_*ij*_ = ∞.
3. If *d*_*j*_ is the same as *f*_*i*_, then *s*_*ij*_ = 0.

For each report, a feature vector, given by [a_1_, a_2_, a_3_, …, a_m_, δ], was generated. δ represents a regularization constant whose value is equal to 10^-k^ where k is a non-negative integer; a_*i*_ ∈. [0,1] represents a vector element associated with the *i*^th^ feature concept and is obtained by the following formula.

1 $$ {\mathrm{a}}_i=\frac{\sqrt{p_i}}{1+\underset{j=1\dots n}{ \min }{s}_{ij}} $$

where *p*_*i*_ is the conditional probability of the *i*^th^ feature concept given the occurrence of HCC. The value of a_*i*_ indicates the relatedness of the *i*^th^ feature concept with the image finding terms of a report. The ability of the feature vector in characterizing a report can be modulated by *p*_*i*_. When the value of *p*_*i*_ is zero, the effect of the *i*^th^ feature concept on the similarity score is fuy repressed. When the value of *p*_*i*_ is one, the effect of the *i*^th^ feature concept on the similarity score is fully promoted.

### Similarity measure

**Fig. 2:**
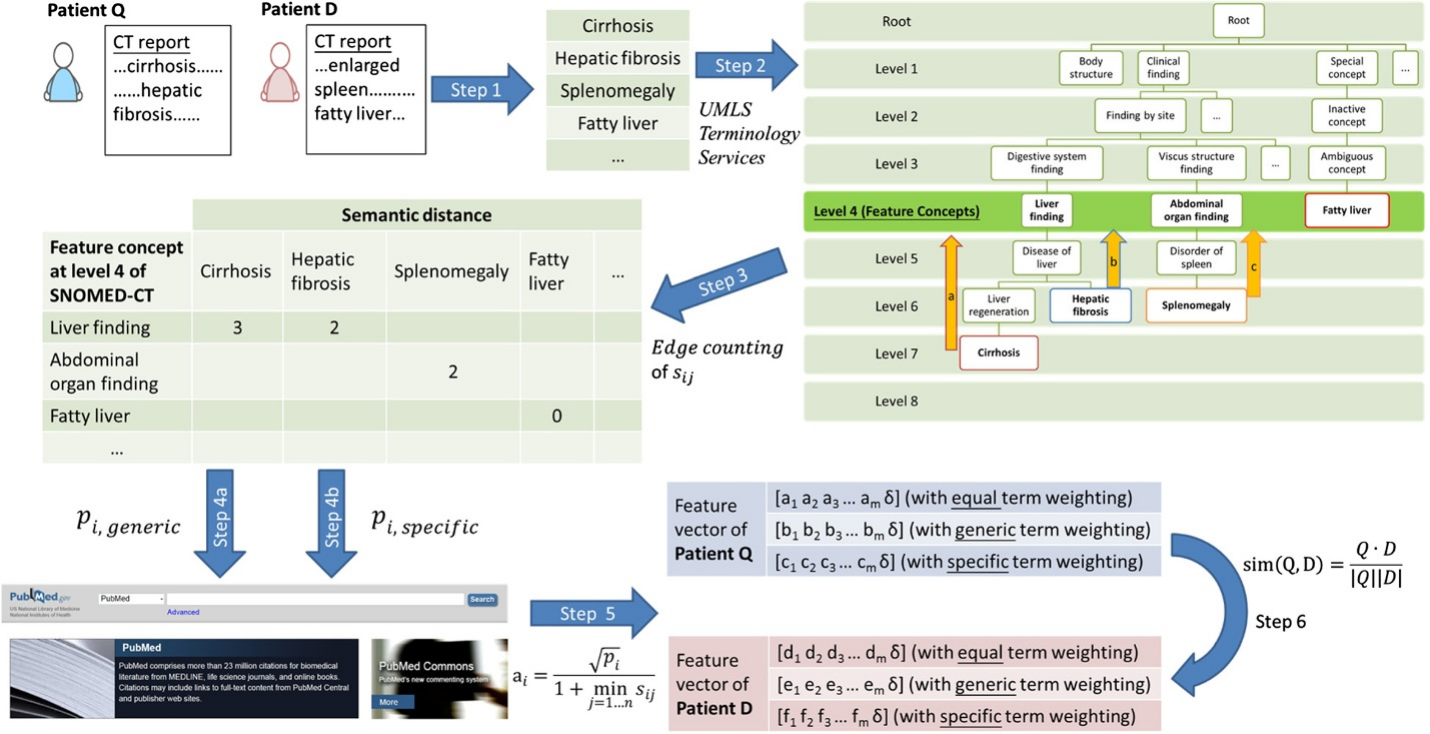
- A schematic view of the method. Step 1: Manual extraction of the image finding terms and their corresponding synonyms from the reports. Step 2: The concepts of the image finding terms defined in SNOMED CT were identified by using UMLS Terminology Services. Step 3: Edge counting of the semantic distances between the extracted terms and the level-4 feature concepts. Step 4: The feature concepts are weighted by (Step 4a) generic term weighting approach and (Step 4b) specific term weighting approach. Step 5: The feature vectors are generated. Step 6: Similarity scores between feature vectors are calculated by modified direction cosine

The similarity score between two reports was calculated by using direction cosine of their feature vectors, Q and D.

2 $$ \mathrm{s}\mathrm{i}\mathrm{m}\left(\mathrm{Q}\kern0.5em \mathrm{D}\right)=\frac{Q\kern0.5em \cdot D}{\left|Q\right|\left|D\right|} $$

where “⋅” is the inner product of two vectors and |*x*|. is the length of a vector *x*. The similarity score ranges from 0 to 1. When the similarity score tends to 0, the vectors Q and D are more dissimilar to each other. When the similarity score tends to 1, they are more similar to each other. To improve inter-patient similarity measure for HCC co-occurrence prediction, this work aims to establish a PubMed-supported approach for estimating more precisely the conditional probability *p*_*i*_. The implementation of the inter-patient HCC co-occurrence prediction is illustrated in Fig. 2.

### Optimization of similarity measure

The similarity measure was optimized by determining its maximum performance in predicting HCC co-occurrence among different values of k. Eqterm weighting (i.e. *p*_*i*_ = 1) is considered as the baseline for the optimization. The choice of k is of crucial importance when the extracted terms are particularly few or even none. If k tends to infinity (δ ≈ 0), the similarity score will be unstable and probably undefined due to the tiny magnitude of feature vector. If k is equal to 0, the similarity score will be dominated by the value of δ irrespective of the reports’ content. The accuracy of the similarity measure in predicting HCC co-occurrence was plotted against the k value. We determined a value of k, at which the accuracy attains maximum according to the trend of the plot. Besides the equal term weighting, the optimal value of k was applied to feature vector for establishing generic and specific term weighting approaches.

### Generic term weighting

According to equation (1), the feature concepts are weighted by a panel of *p*_*i*_, which is defined as the conditional probability of the *i*^th^ feature concept given HCC. Literature search was performed by using PubMed and the numbers of abstracts listed in the search results were used for the estimation of *p*_*i*_. Generic term weighting was implemented by applying directly the following formula.

3 $$ {p}_{i,\  generic}=\frac{\#\ \mathrm{of}\ \mathrm{abstracts}\ \mathrm{containing}\ \left[\left(A\ \mathrm{OR}\ {A}_1^{\hbox{'}}\ \mathrm{OR}\ {A}_2^{\hbox{'}}\ \mathrm{OR}\dots \right)\ \mathrm{AND}\ \left(B\ \mathrm{OR}\ {B}_1^{\hbox{'}}\ \mathrm{OR}\ {B}_2^{\hbox{'}}\ \mathrm{OR}\dots \right)\right]}{\#\ \mathrm{of}\ \mathrm{abstracts}\ \mathrm{containing}\ \left(B\ \mathrm{OR}\ {B}_1^{\hbox{'}}\ \mathrm{OR}\ {B}_2^{\hbox{'}}\ \mathrm{OR}\dots \right)} $$

where *A* and *A*_*n*_^'^ are the *i*^th^ feature concept and its *n*^th^ synonym respectively; *B* and *B*_*n*_^'^ represent HCC and its *n*^th^ synonym respectively. Using this approach, the calculated weights of feature concepts were the same among different reports although their descendent terms extracted from the reports are different.

### Specific term weighting

In specific term weighting approach, we seched PubMed for the abstracts containing the extracted terms and HCC. The conditional probability of the *m*^th^ extracted term given HCC, *q*_*m*_, were estimated by the following formula.

4 $$ {q}_m=\frac{\#\kern0.5em \mathrm{of}\kern0.5em \mathrm{abstracts}\kern0.5em \mathrm{containing}\kern0.5em \left[\left(C\kern0.5em \mathrm{OR}\kern0.5em {C}_1^{\mathit{\hbox{'}}}\kern0.5em \mathrm{OR}\kern0.5em {C}_2^{\mathit{\hbox{'}}}\kern0.5em \mathrm{OR}\dots \right)\kern0.5em \mathrm{AND}\kern0.5em \left(B\kern0.5em \mathrm{OR}\kern0.5em {B}_1^{\mathit{\hbox{'}}}\kern0.5em \mathrm{OR}\kern0.5em {B}_2^{\mathit{\hbox{'}}}\kern0.5em \mathrm{OR}\dots \right)\right]}{\#\kern0.5em \mathrm{of}\kern0.5em \mathrm{abstracts}\kern0.5em \mathrm{containing}\kern0.75em \left(B\kern0.5em \mathrm{OR}\kern0.5em {B}_1^{\mathit{\hbox{'}}}\kern0.5em \mathrm{OR}\kern0.5em {B}_2^{\mathit{\hbox{'}}}\kern0.5em \mathrm{OR}\dots \right)} $$

where *C* and *C*_*n*_^'^. are the *m*^th^ extracted term and its *n*^th^ synonym respectively; *B* and *B*_*n*_^'^ represent HCC and its *n*^th^ synonym respectively. We assumed that the conditional probability of the *i*^th^ feature concept given HCC is equal to the average of the conditional probabilities of its *N* descendent terms extracted from a report given HCC. The value of *p*_*i*_ was calculated by the following formula.

5 $$ {p}_{i,\kern0.5em  specific}=\frac{q_1+{q}_2+{q}_3+\dots }{N} $$

Note that the weighting of feature vector elements is dependent of the report content. In contrast to the generic term weighting where the weights don’t change across reports, the weights of the same feature concept estimated by specific term weighting approach may differ from patient to patient.

### Statistical analysis

The receiver operating characteristic (ROC) analysis was performed to the results of inter-patient HCC co-occurrence predicted by equal, generic and specific term weighting approaches. For each approach, the ROC curve was plotted and the Areas under the ROC curve (AUROC) indicated the accuracy of the prediction, i.e. the probability of correctly classifying a pair of reports into same diagnosis (both are HCC; both are NAD) or different diagnosis (one is HCC and the other is NAD). In addition to the comparison with the area under the chance diagonal, AUROCs were compared with each other to determine an approach with the best performance and the statistical significance of the observed differences were also indicated [17, 18].

## Results

### Feature extraction and report pair formation

We extracted 38 image finding terms from 112 examination reports (59 HCC and 53 NAD cases). These terms are uniquely defined by 38 concepts in UMLS and were projected to 36 feature concepts at level-4 of SNOMED CT “is-a” hierarchy. The reports were paired up to form 6216 non-redundant pairs, in which 3089 pairs are matches, i.e. (HCC,HCC) or (NAD,NAD), and 3127 pairs are mismatches, i.e. (HCC,NAD) or (NAD,HCC).

### Optimization of similarity measure

**Fig. 3:**
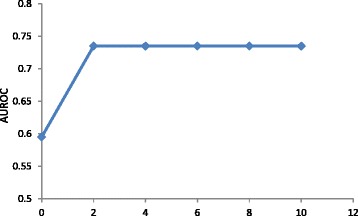
- Plot of AUROC against the value of k. The accuracy of inter-patient HCC co-occurrence prediction increases when k is between 0 and 2 and saturates at the level of 0.735 when k further increases

Equal term weighting was considered as baseline for optimizing the similarity measure. ROC analysis of inter-patient HCC co-occurrence prediction was performed for different values of k. Fig. 3 shows the plot of AUROC against k. It was found that the accuracy increases for k between 0 and 2. For k > 2, the AUROC reaches a constant level. Thus, we chose k = 10 for all the term weighting approaches.

### Estimation of conditional probabilities

In generic term weighting, abstracts were retrieved by PubMed search for each feature concept and its synonyms. The count of abstracts containing a feature concept or its synonyms ranges from 1 to 427154. By incorporating HCC and its synonyms to the search criteria, the abstract count was further reduced. The conditional probability of a feature concept for generic term weighting is defined as the ratio of these two counts.

In specific term weighting, abstracts were retrieved by PubMed search for each extracted term and its synonyms. The count of abstracts containing a feature concept or its synonyms ranges from 1 to 195708. The abstract count is further reduced by adding HCC and its synonyms to the search criteria. The ratio of these two counts was projected to the corresponding feature concepts. The conditional probability of a feature concept for specific term weighting is defined as the average of the ratios across all of its descendent terms extracted from a report. The values of conditional probabilities were computed and saved in Excel files (See Additional files 1, 2, 3, 4).

### Comparison of term weighting approaches

The AUROCs and the 95 % confidence intervals (95 % CIs) of equal, generic and specific term weighting approaches are shown in Table 1. It was found all three approaches outperformed the random rater significantly (p < 0.01). When compared to equal term weighing approach (AUROC = 0.735), the performance was significantly improved by specific term weighting approach (AUROC = 0.743, p < 0.01) but was significantly worsen by generic term weighting approach (AUROC = 0.728, p < 0.01). The conditional probabilities of the extracted image finding terms given HCC, derived by the specific term weighting approach, were sorted in descending order. The top ten image finding terms are listed together with their conditional probabilities in Table 2.

**Table 1:** - Comparison of term weighting approaches. AUROCs and the 95 % CIs of the equal, generic and specific term weighting approaches are summarized here

Term weighting approach	AUROC	95 % CI
Equal term weighting	0.735	(0.724, 0.746)
Generic term weighting	0.728	(0.717, 0.739)
Specific term weighting	0.743	(0.732, 0.754)

**Table 2:** - Top ten image finding terms. The PubMed search results indicated that some image finding terms were co-mentioned with HCC very frequently in the abstracts of biomedical journal articles. The conditional probability of “Dysplastic nodule” (0.934) is the highest among all the extracted terms

Rank	Image finding	Conditional probability
1	Dysplastic nodule	0.934
2	Nodule of liver	0.513
3	Equal density (isodense) lesion	0.438
4	Nodular hyperplasia of liver	0.329
5	Solitary necrotic liver nodule	0.259
6	Portal vein thrombosis	0.209
7	Space occupying lesion of liver	0.175
8	Cirrhosis of liver	0.170
9	Hepatic fibrosis	0.082
10	Nontraumatic hemoperitoneum	0.064

## Discussion

Health records ontologically similar to new suspected case support clinical decision with evidence of the disease. The reliability of such ontology-similarity-based case retrieval algorithm depends on the choices of inter-patient similarity measure and ontological vector model. It has been proved that modified Direction Cosine (mDC) avoids the problem of numerical overflow and preserves the same properties as Euclidean distance does [12]. However, weighting of the ontological vector was not considered in the previous studies and it remained unknown if the performance of mDC can be improved by adjusting the weights associated with the feature concepts. It was shown that the performance of the similarity measure was substantially improved by setting an extremely small regularization constant, 10^−10^. Such setting helps maintain the similarity scores discriminative for comparing health records that have very few or even no extracted clinical terms.

In generic term weighting, the median and the 10th percentile of the counts of retrieved abstracts containing the feature concepts or their synonyms are 4204 and 82.9 respectively. In specific term weighting, the median and the 10th percentile of the counts of retrieved abstracts containing the extracted terms or their synonyms are 2689.5 and 46.5 respectively. The sample sizes of the retrieved abstracts are large enough to support the estimation of conditional probabilities.

In comparison to equal term weighting, the performance was improved by specific term weighting approach but worsened by generic term weighting approach. It implied that the weighted feature vector elements do not necessarily give better performance but the way, through which we derived the weights, is crucial for improving performance. In generic term weighting, the feature concepts at level-4, instead of the clinical terms extracted from the reports, were used for PubMed search. The weights are associated with the level-4 concepts only and remain unchanged across different reports. Moreover, the level-4 concepts are not specific enough to provide reliable results of PubMed search for estimating the conditional probabilities. Specific term weighting used the extracted terms directly for PubMed Search. The search results are more reliable for estimating the conditional probabilities due to the higher granularity of concepts provided by the extracted terms. Although the conditional probabilities of the descendent extracted terms are averaged to generate the weights of feature vector elements, the keywords for PubMed Search are dynamically dependent of the report contents and the weights become more specific.

The high weights of feature concepts dominating the similarity score are attributed by their descendent terms extracted from the reports. In Table 2, the top three image finding terms (conditional probabilities) are “dysplastic nodule” (0.934), “nodule of liver” (0.513) and “equal density (isodense) lesion” (0.438). The association of these image findings with HCC is supported by Sakamoto [19] stating that small equivocal lesions, i.e. dysplastic nodules, detected by imaging examination of liver are regarded as a precursor of HCC. For the cases with such image finding but no abnormality detected, we suggest to index them as “high risk” so that close follow-up can be recommended to those patients.

As the conditional probability of the most relevant image finding “Dysplastic nodule” (0.934) is greater than ten times of that of the eighth image finding “Hepatic fibrosis”, 0.082, only the top seven features are significantly contributed to the diagnosis prediction performance. The features other than these top seven features are associated with negligible weights and have negligible effect on the prediction. Therefore, the generic and specific term weighting approaches are analogous with feature selection that makes the vector model more parsimonious with respect to the number of available cases.

As the numbers of PubMed abstracts are dynamic, the term weighting results may change from time to time. In our future studies, it is suggested to enhance the ontological vector model by incorporating more algorithmic elements from information content model, which spans an essential dimension of assessing the semantic similarity [20].

Besides the image examination report, laboratory test findings, such as Alpha fetoprotein (AFP) level and Child-Pugh score, are important features for the diagnosis of HCC. As the electronic health record (EHR) integrated the image examination report and laboratory test report, the feature vector can be augmented to cover laboratory test findings [21]. The same weighting approach and similarity measure can also be applied to such augmented feature vector.

Our development of clinical term weighting approach not only improved the inter-patient similarity measures for diagnosis prediction. In fact, this method may be used to identify large cohorts of patients with similar disease presentation for retrospective treatment efficacy analysis. It may also facilitate the identification of targeted patient cohorts for prospective interventional studies.

## Conclusions

The performance of inter-patient similarity measure was significantly improved by specifically weighting the elements of the ontological feature vector. PubMed search was applied to estimate the weights. Early HCC markers, including dysplastic nodule, nodule of liver, and equal density lesion, were identified by PubMed search as image findings that are strongly associated with HCC.

## Additional files

Additional file 1: **Generic Term Weighting Computation.**

Additional file 2: **Specific Term Weighting Computation.**

Additional file 3: **Similarity Scores with Generic Term Weighting.**

Additional file 4: **Similarity Scores with Specific Term Weighting.**
